# Antiplatelet Agents for Cancer Prevention: Current Evidences and Continuing Controversies

**DOI:** 10.3390/cancers11111639

**Published:** 2019-10-24

**Authors:** Corinne Frere, Manon Lejeune, Pierre Kubicek, Dorothée Faille, Zora Marjanovic, the Groupe Francophone Thrombose et Cancer

**Affiliations:** 1 Sorbonne Université, GRC 27 GRECO, F-75013 Paris, France; 2 INSERM UMRS_1166, Institute of Cardiometabolism and Nutrition, F-75013 Paris, France; 3 Department of Haematology, Pitié-Salpêtrière Hospital, Assistance Publique Hôpitaux de Paris, F-75013 Paris, France; lejeune.manon@gmail.com; 4 Institut de cancérologie de l’Ouest (ICO), Site Paul Papin, 49055 Angers, France; pier220390@hotmail.fr; 5 Université de Paris, INSERM UMRS_1148, F-75018 Paris, France; dorothee.faille@aphp.fr; 6 Laboratory of Haematology, Bichat Hospital, Assistance Publique Hôpitaux de Paris, F-75018 Paris, France; 7 Department of Haematology, Saint-Antoine Hospital, Assistance Publique Hôpitaux de Paris, F-75012 Paris, France; zora.marjanovic@aphp.fr

**Keywords:** antiplatelet drugs, colorectal cancer, cancer, cancer-related mortality, adenoma, Lynch syndrome, chemoprevention, aspirin, thienopyridines

## Abstract

Over the past two decades, aspirin has emerged as a promising chemoprotective agent to prevent colorectal cancer (CRC). In 2016, the mounting evidence supporting its chemoprotective effect, from both basic science and clinical research, led the US Preventive Services Task Force to recommend regular use of low-dose aspirin in some subgroups of patients for whom the benefits are deemed to outweigh the risks. In contrast, data on the chemoprotective effect of aspirin against other cancers are less clear and remain controversial. Most data come from secondary analyses of cardiovascular prevention trials, with only a limited number reporting cancer outcomes as a prespecified endpoint, and overall unclear findings. Moreover, the potential chemoprotective effect of aspirin against other cancers has been recently questioned with the publication of 3 long-awaited trials of aspirin in the primary prevention of cardiovascular diseases reporting no benefit of aspirin on overall cancer incidence and cancer-related mortality. Data on the chemoprotective effects of other antiplatelet agents remain scarce and inconclusive, and further research to examine their benefit are warranted. In this narrative review, we summarize current clinical evidence and continuing controversies on the potential chemoprotective properties of antiplatelet agents against cancer.

## 1. Introduction

Over the past two decades, there has been mounting evidence, from both basic science and clinical research, supporting a chemoprotective effect of aspirin against colorectal cancer (CRC) [1,2,3,4,5,6,7]. In 2016, this large body of evidence led the U.S. Preventive Services Task Force (USPSTF) to recommend regular use of low-dose aspirin to prevent CRC in some subgroups of patients for whom the benefits are deemed to outweigh the risks (patients aged 50 to 69 years who have a low risk of bleeding). [8] Aspirin is also recommended by the US Multi-society Task Force on Colorectal Cancer [9] and the National Institute for Health and Care Excellence [10] to prevent CRC in high risk patients with Lynch syndrome.

In contrast, data on the chemoprotective effect of aspirin against other cancers are less clear and remain controversial. One major limiting factor is that most data come from secondary analyses of cardiovascular prevention trials, with only a limited number reporting cancer outcomes as a prespecified endpoint, and overall unclear findings. Moreover, the potential chemoprotective effect of aspirin against cancers other than CRC has recently been questioned with the publication of 3 long-awaited trials of aspirin in the primary prevention of cardiovascular diseases (CVD) reporting no benefit of aspirin on overall cancer incidence and cancer-related mortality [11,12,13].

The mechanism of action by which aspirin exerts its anticancer effect is not fully understood, but it has been proposed that it is mediated by its antiplatelet properties and extensively reviewed elsewhere [2,3,5,6,7].

Data on the chemoprotective effects of other antiplatelet agents remain scarce and inconclusive, and further research to examine their benefit are warranted.

Here, we review current clinical evidence and continuing controversies on the potential chemoprotective properties of antiplatelet agents against cancer.

## 2. Chemoprotective Effect of Aspirin

### 2.1. Chemoprotective Effect of Aspirin on Colorectal Cancer

#### 2.1.1. General Population

##### Evidence from Epidemiological Studies

The inverse association between aspirin use and the risk of colorectal cancer (CRC) was first reported in a population-based case-control study in 1988. This study aimed to investigate the potential protective effects of aspirin and found a 47% risk reduction of CRC in patients taking aspirin, or aspirin-containing medication (Relative Risk (RR), 0.53; 95% confidence interval (CI), 0.4–0.71; *p* < 0.001) [14]. Numerous prospective cohorts or case-control studies have since corroborated these findings, consistently reporting the protective effects of aspirin on the incidence of CRC, when given as a long-term treatment once per day at low doses (75–500 mg per day). In the Cancer Prevention Study II (CPS II) Nutrition Cohort, a large prospective study of cancer incidence and mortality (*n* = 146,113 participants), long-term daily aspirin use (for 5 years or more) was associated with a significantly lower incidence of CRC among both men and women (RR, 0.68, 95% CI, 0.52–0.90) [15]. Similarly, a large Danish population-based case-control study (10,280 CRC cases and 102,800 controls) reported a significantly decreased risk of CRC in patients who consecutively filed low-dose aspirin prescriptions for more than 5 years (Odds Ratio(OR), 0.73; 95%, CI 0.54–0.99) [16].

More recently, Cao et al. assessed the potential benefits of aspirin for cancer prevention based on 2 large prospective US cohort studies. [17] In their combined cohort analysis, regular aspirin use was associated with a slight (3%) reduction in the risk of cancer overall (*n* = 135,965; RR, 0.97; 95% CI, 0.94–0.99). Importantly, this reduction was mainly driven by a significant 15% reduction in the risk of gastrointestinal tract cancers (RR, 0.85; 95% CI, 0.80–0.91), especially CRC (RR, 0.81; 95% CI, 0.75–0.88). A lower risk was observed only for a duration of aspirin treatment longer than 6-years. There was no association between regular use of aspirin and the risk for breast, advanced prostate, or lung cancer [17]. The chemoprotective effect of aspirin on gastrointestinal tract cancers emerged at a dose of 0.5 to 1.5 standard aspirin tablets per week or the equivalent of a daily dose, and was dose-dependent, with significantly greater effects observed with increasing doses. Interestingly, Cao et al. also reported on the partial population attributable risk in patients older than 50: Regular aspirin use could have prevented 33 CRC per 100,000 person-years (17.0%) among subjects who had not undergone a lower endoscopy and 18 CRC per 100,000 person-years (8.5%) among those who had undergone a lower endoscopy [17,18]. These findings also suggest that aspirin may have an absolute benefit regardless of whether the patient has received endoscopy screening.

##### Evidence from Post-Hoc Analyses of Randomized Controlled Trials Assessing the Efficacy and Safety of Aspirin in the Prevention of Cardiovascular Disease

Compelling evidence for the effects of aspirin on reduction of the incidence of CRC has also been provided by post-hoc analyses of randomized controlled trials (RCTs) that were mainly designed to assess the safety and efficacy of aspirin in the primary or secondary prevention of CVD. (Table 1) Few RCTs have reported cancer outcomes as a prespecified endpoint. Post-hoc analyses reached conflicting findings. The Physician Health Study (PHS), a randomized, double-blind, placebo-controlled trial of alternate-day doses of aspirin (325 mg) versus placebo [19], and the Women’s Health Study (WHS) [20], a randomized, factorial, placebo-controlled trial of alternate-day doses of aspirin (100 mg) versus placebo, did not find any reduction in CRC incidence over a 10 years follow-up period (RR, 1.03; 95% CI, 0.83–1.28 in PHS and RR, 0.97; 95% CI, 0.77–1.24 in WHS). However, in an extended follow-up analysis of the WHS, a significant 42% reduction in the incidence of CRC was observed in the aspirin group after 10 years (RR,0.58; 95% CI, 0.42–0.80) [21].

In 2007, the first individual patient data (IPD) meta-analysis that pooled results from two large RCTs [22,23], reported an overall 37% reduction in CRC incidence in aspirin users treated for five years or more (pooled Hazard Ratio (HR), 0.63, 95% CI, 0.47–0.85; *p* = 0.002). As in the WHS, the significant benefit of aspirin use was observed only 10 years after randomization [24]. This result was subsequently confirmed in another IPD meta-analysis of four pooled trials investigating long-term administration of low-dose aspirin (75–300 mg per day) which reported a 24% reduction in the 20-year risk of CRC (*n* = 14,033; HR, 0.76; 95% CI, 0.63–0.94) [25]. The benefit of aspirin use increased with treatment beyond five years (HR, 0.68; 95% CI, 0.54–0.87). The PHS and WHS were not included in this meta-analysis, but the results are consistent with those of the extended follow-up analysis of the WHS [21]. Similarly, a systematic review of the literature conducted to support the USPSTF evidence-based recommendations on the use of aspirin for primary prevention of CRC found a 40% reduction in CRC risk after 10 to 19 years of initiation of aspirin treatment (RR, 0.60; 95% CI, 0.47–0.76) [26]. Numerous further meta-analyses including both observational and randomized controlled studies have also consistently reported that long-term aspirin use is associated with a 30–40% CRC risk, albeit with minor heterogeneity observed between studies [27,28,29].

Both epidemiological studies and meta-analyses of RCT have also provided evidence for the benefit of aspirin on CRC-related mortality. In the CPS II study, CRC-related mortality was decreased in participants using aspirin 16 or more times per month, for at least one year (men: RR, 0.60; 95% CI, 0.40–0.89; women: RR, 0.58; 95% CI, 0.37–0.90) [30]. In a meta-analysis of the long-term effects of aspirin on CRC, Rothwell et al. found that the 20-year cumulative risk of CRC-related death was significantly decreased in patients taking aspirin on a daily basis (RR, 0.65; 95% CI, 0.48–0.88), with improved benefits observed in those taking daily aspirin for at least 5 years (RR, 0.63; 95% CI, 0.45–0.87) [25].

In 2016, the USPSTF reviewed both the beneficial and harmful effects of aspirin in the primary prevention of CVD and CRC, along with a decision analysis to support their position statement [8,26,31,32]. The large body of available evidence was considered to be strong enough to recommend aspirin for primary prevention of CVD and CRC in patients aged 50 to 59 years with a low risk of bleeding and a 10-year atherosclerotic CVD risk of 10% (B recommendation). In patients aged 60 to 69 years meeting this risk threshold, the USPSTF recommends aspirin use to be considered through shared decision-making (C recommendation) [8]. For patients younger than 50 years of age, or older than 70, the evidence was considered to be inconclusive [8].

#### 2.1.2. Patients at High-Risk of CRC

The beneficial effects of aspirin were also widely investigated in individuals at high-risk of CRC. Colorectal adenoma represents the most frequent precancerous lesion for CRC. Effective chemoprevention, in addition to screening and surveillance programs, is therefore warranted in patients with previous resected colorectal adenoma. Several placebo-controlled trials investigating aspirin for chemoprevention of recurrent adenoma and CRC were conducted over the past number of years, but they have reported conflicting results (Table 2).

A meta-analysis of four randomized double-blind placebo-controlled trials [33,34,35,36] evaluating the efficacy and safety of aspirin (75 to 325 mg daily) for the prevention of recurrent colorectal adenomas included 2,967 patients with a previous history of colorectal adenoma or CRC. The pooled analysis of these trials found a statistically significant 17% decrease in the relative risk of recurrent adenoma in patients receiving aspirin, at any dose, versus patients receiving placebo (pooled RR, 0.83; 95% CI, 0.72–0.96), and a 28% decrease in the relative risk of advanced lesions (pooled RR, 0.72; 95% CI, 0.57–0.90) [37]. The chemoprotective effect of aspirin was not found to differ between high and low-dose aspirin, for any adenomas or for advanced lesions [37]. Similarly, the randomized, placebo-controlled multicenter Japan Colorectal Aspirin Polyps Prevention (J-CAPP) trial reported a lower risk of any recurrent adenoma or CRC in the aspirin arm compared to the placebo arm (*n* = 311; RR, 0.60; 95% CI, 0.36–0.98) [38].

In contrast, a large randomized, double-blind, placebo-controlled trial found no overall association between treatment with a combination of 75 mg aspirin, calcitriol and calcium carbonate, and recurrent adenoma (*n* = 1,107; OR, 0.95; 95% CI, 0.61–1.48). This trial was stopped prematurely because of its negative results. A high dropout rate and low compliance was noticed. However, a trend toward a decrease in risk was observed in the aspirin treatment group in non-smokers compared to smokers (OR, 0.65; 95% CI, 0.26–1.22) [39]. Consistent with this finding, a subgroup analysis of the J-CAPP trial revealed that the benefit of aspirin use was confined to non-smokers (OR, 0.37; 95% CI, 0.21–0.68; *p* = 0.01 in non-smokers versus OR, 3.45; 95% CI, 1.12–10.64; *p* = 0.03 in smokers) [38]. Additional data on modifiers of aspirin benefit in smokers are warranted. None of these studies reported an association between aspirin and CRC-related mortality.

Recently, the multicenter, randomized, double-blind, placebo-controlled, 2 × 2 factorial SeAFOod Polyp Prevention trial [40] assessed the efficacy of aspirin and eicosapentaenoic acid (EPA) in preventing colorectal adenoma in patients included in the English Bowel Cancer Screening Programme and identified as being at high risk during colonoscopy. Seven hundred and nine patients were randomized to receive either 300 mg aspirin per day, 2 g EPA per day, a combination of both, or placebo for 12 months. The primary end point of adenoma rate did not differ between groups. Aspirin use was not associated with a decreased rate of adenoma (RR, 0.99; 95% CI, 0.87–1.12; *p* = 0.88). However, a significant decrease in the total mean number of colorectal adenomas per patient was observed, as well as a decreased incidence of conventional colorectal adenomas, and a decreased incidence rate ratio of right-sided and serrated lesions [40]. No CRC was detected during a one-year follow-up [40].

Taken together, these data indicate a small but significant benefit of aspirin in preventing adenoma and CRC and suggest the need for an individualized rather than a one-size-fits-all approach to CRC chemoprevention.

Lynch syndrome, or hereditary nonpolyposis colorectal cancer, is an autosomal dominant inherited cancer-prone syndrome due to germline loss-of-function mutations in the *MLH1, MSH2, MSH6, and PMS2* genes. It is the most common cause of inherited CRC, accounting for approximatively 3% of all cases [9,43]. Patients with Lynch syndrome have a life-time risk of CRC of up to 74% [9] and a significantly increased risk for a wide variety of other cancers including endometrial, ovary, stomach, small intestine, liver, gallbladder ducts, upper urinary tract, and brain. [9,43] Synchronous and metachronous cancers are frequent, up to 41% at 20 years [9,43]. The Colorectal Adenoma/Carcinoma Prevention Programme 2 (CAPP2) randomized, double-blind, placebo-controlled trial assessed the efficacy and safety of aspirin and resistant starch on the development of colorectal adenoma and cancer. The trial included 937 patients who carry a Lynch syndrome pathologic mismatch-repair mutation. Patients were randomly assigned to receive either high-dose aspirin (600 mg per day) plus resistant starch placebo, resistant starch (30 g) plus aspirin placebo, aspirin plus resistant starch, or aspirin placebo plus starch placebo, for up to 4 years. A first pre-planned analysis reported no reduction in the risk of colorectal adenoma or carcinoma after a mean follow-up of 29 months in patients receiving 600 mg aspirin daily (RR,1.00; 95% CI, 0.7–1.4) [44]. However, a non-significant reduction in the rate of colorectal neoplasia was observed in the pooled aspirin groups compared to the pooled placebo groups (8.4% versus 10.9%; *p* = 0.3) [44]. In a second pre-planned analysis, conducted after the first participants reached the 10 year follow-up, while the intention to-treat analysis rates of CRC tended to be lower in the patients receiving aspirin (3.8% versus 8.2% in patients receiving aspirin placebo; HR, 0.41; 95% CI, 0.35–1.13; *p* = 0.12), aspirin was associated with a significant 39% decrease in the relative risk of CRC in patients who had completed two years of intervention and after a mean follow-up of 55·7 months (HR, 0.41; 95% CI, 0.19–0.86; *p* = 0.02) [45], suggesting a delayed benefit of aspirin, as observed in previous observational studies and meta-analyses. Furthermore, aspirin seemed to confer a protective effect from cancers (i.e., colorectal, endometrial, ovarian, pancreatic, small bowel, gallbladder, ureter, stomach, kidney, and brain; HR, 0.63; 95% CI, 0.34–1.19; *p* = 0.16) [45]. Importantly, the CAPP2 trial found no differences in the occurrence of adverse events between aspirin and placebo groups. Moreover, a retrospective cohort study of patients with Lynch syndrome who are carriers of a pathogenic germline mutation in the MLH1, MSH2, MSH6, or PMS2 gene reported that aspirin use was associated with a decreased risk of colorectal cancer (for 1 month to 4.9 years: HR, 0.49, 95% C, 0.27–0.90; for ≥ 5 years: HR, 0.25; 95% CI, 0.10–0.62) [46].

Based on these finding, since 2014 the United States Multi-society Task Force on CRC has recommended that treatment with aspirin should be considered in individual patients with Lynch syndrome after the patient-specific risks, benefits, and uncertainties of treatment have been discussed [9]. More recently, the National Institute for Health and Care Excellence (NICE) has recommended that routine use of aspirin should be considered for the prevention of CRC in patients with Lynch syndrome, at daily doses of 600 mg or 300 mg or 100 mg, and for a duration longer than two years. NICE also recommends that informed consent should be obtained from the patient for prescribing decisions, and that the process should be documented since aspirin has not yet been approved for CRC prevention [10].

The optimal dose and duration of aspirin treatment for preventing Lynch syndrome cancers remains unknown. The ongoing Colorectal Adenoma/Carcinoma Prevention Programme 3 (CAPP3) double-blind dose, non-inferiority trial will randomize patients with Lynch Syndrome to receive daily doses of 600 mg, 300 mg, or 100 mg of enteric coated aspirin for two years. The study primary endpoint is the incidence of Lynch Syndrome cancers throughout the study and during 10 years of follow-up (ISRCTN16261285).

### 2.2. Chemoprotective Effect of Aspirin on Other Cancers

#### 2.2.1. Overall Cancer Incidence

The protective effect of aspirin against other cancers is much less clear. While observational studies have suggested that aspirin might also reduce the incidence of cancer of the esophagus [47,48,49,50] and stomach [47,49,51,52], its benefit in reducing the incidence of other cancers such as lung [15,17,53,54,55] and breast cancers [56,57,58,59] appears less consistent.

A time-to-event IPD meta-analysis of 6 trials assessing daily low-dose aspirin in primary cancer prevention reported a slight reduction in overall cancer incidence in aspirin users compared to aspirin non-users more than three years after randomization, regardless of sex (*n* = 35,535; OR, 0.76; 95% CI, 0.66–0.88; *p* = 0.0003) [60]. However, a systematic review re-analyzing this set of trials found no significant effect of aspirin on overall cancer incidence after exclusion of two of the studies reporting on fatal cancers only and providing concomitant treatment with vitamin K antagonists (4 trials; RR, 0.92; 95% CI, 0.82–1.02) [26]. Aspirin did not reduce cancer rates in six primary prevention trials with a follow-up duration ranging from 3.6 to 10.1 years (*n* = 72,926; RR, 0.98; 95% CI, 0.93) [26]. A marginally significant decrease in cancer incidence was observed only when the analysis was restricted to trials of daily aspirin use, including secondary prevention studies, and having a median intended duration of at least four years (four trials; *n* = 11,800; RR, 0.86; 95% CI, 0.74–0.99) [26].

The large WHS study found no reduction in the incidence of any cancer among aspirin users during an average 10 year follow-up (*n* = 39,876; RR, 1.01; 95% CI, 0.94–1.08; *p* = 0.87) [20]. An extended follow-up analysis (median, 17.5 years; range, 10.4–18.8 years) yielded similar results; aspirin had no effect on the incidence of total (HR, 0.97; CI, 0.92–1.03), breast (HR, 0.98, 95% CI, 0.90–1.07), and lung cancer (HR, 1.04, 95% CI, 0.86–1.26), either over the entire follow-up, the within-trial, or post-trial follow-up periods [21]. A significant reduction in incident cancer was observed in aspirin users only for CRC (HR, 0.80, 95% CI, 0.67–0.97) [21].

Moreover, the chemoprotective effects of aspirin on overall cancer incidence have been recently questioned with the publication of long-awaited large clinical trials assessing the efficacy of aspirin in the primary prevention of CVD. The ASCEND (A Study of Cardiovascular Events iN Diabetes) trial, which randomly assigned 15,480 patients with diabetes to receive either 100 mg aspirin or placebo daily, reported no difference in the incidence of any cancer within a mean follow-up of 7.4 years (rate ratio, 1.01, 95% CI, 0.92–1.11) [11]. Longer-term follow-up is ongoing to evaluate if any effects on cancer emerge later as in the extended follow-up analysis of the WHS. The Aspirin to Reduce Risk of Initial Vascular Events (ARRIVE) trial randomized 12,456 patients (aged 55 to 60 years) with moderate CV risk and without diabetes to receive 100 mg aspirin daily or placebo for 5 years. Within a median follow-up of 60 months, few cancer outcomes were reported (0.94% of prostate cancers in aspirin users versus 0.72% in aspirin non-users; 0.22% of CRC in aspirin users versus 0.70% in aspirin non-users), without any differences between groups [12]. Finally, a 2019 Bayesian meta-analysis of 13 RCTs including 10 RCTs reporting overall cancer incidence found no difference in the rates of incident cancers between aspirin users and aspirin non-users (HR, 1.01; 95% CI, 0.93–1.08) [61].

#### 2.2.2. Overall Cancer-Related Mortality

Five meta-analyses of RCTs comparing daily aspirin versus no aspirin use in the prevention of CVD or in any population of patients treated with aspirin [60,61,62,63,64] and a systematic review of the literature for the USPSTF [26] have assessed the association between aspirin and overall cancer-related mortality, with conflicting results. While three meta-analyses suggested that daily aspirin use over a five-year treatment period or more might reduce the risk of cancer-related mortality [60,62,64], two others reported no benefit [61,63].

In an early IPD meta-analysis including eight RCTs comparing daily aspirin versus no aspirin use in the primary or secondary prevention of CVD, daily aspirin use resulted in a 21% reduction in the risk of cancer-related mortality (*n* = 25,570 patients; OR, 0.79; 95% CI, 0.68–0.92, *p* = 0.003) [62]. When analyzing individual patient data (7 RCTs), the benefit of aspirin use on cancer-related mortality was observed regardless of dose, but only after 5 or more years of follow-up (HR, 0.66; 95% CI, 0.50–0.87 for all cancers and HR, 0.46; 95% CI, 0.27–0.77 for gastrointestinal cancers; *p* = 0.003 for both). This beneficial effect increased with treatment duration, for up to 20 years. For lung and esophageal cancers, the benefit of aspirin use was restricted to adenocarcinoma (HR, 0.66; 95% CI, 0.56–0.77; *p* < 0.001). The greatest absolute risk reduction occurred in patients older than 65 years after 20 years of follow-up [62]. A second meta-analysis (69,224 patients) by the same authors corroborated these finding; an inverse association between daily aspirin use and the risk of cancer-related mortality was observed (in 34 RCTs; OR, 0.85; 95% CI, 0.76–0.96; *p* = 0.008), with a 37% reduction in the risk of cancer-related mortality in aspirin users after a treatment duration of 5 years or more (OR, 0.63, 95% CI 0.49–0.82; *p* = 0.0005) [60]. A third meta-analysis included eleven RCTs comparing daily aspirin versus no aspirin in any population (41,398 patients). Daily aspirin use after at least 2.5 years of follow-up was associated with a 23% reduction in the risk of cancer-related mortality (RR, 0.77; 95% CI, 0.63–0.95) [64].

In contrast, a pooled analysis of eight RCTs comparing daily aspirin versus placebo in the primary prevention of CVD found no significant reduction in cancer-related mortality (OR, 0.93; 95% CI, 0.84–1.03) [63] Similarly, in the Chubak et al. review, when analyses were restricted to primary prevention of CVD trials, aspirin use was not found to decrease the risk of cancer-related mortality over 3.7 to 10.1 years (RR, 0.96; 95% CI, 0.87–1.06) [26]. However, when including trials of both primary and secondary prevention of CVD with a treatment duration of 4 years or more, aspirin use was associated with slightly significant benefit as regards cancer mortality benefit (RR, 0.83; 95% CI, 0.70–0.98) [26], as previously reported [60,62,63,64].

Of note, while earlier meta-analyses found that the reduction in cancer-related mortality emerged after at least five years of follow-up in aspirin users, this finding was not replicated in the large ARRIVE trial which did not find any difference in cancer-related-mortality between aspirin users and aspirin non-users despite a mean follow-up of 7.4 years (rate ratio, 0.98; 95% CI, 0.84–1.15).

Recently, results from the Aspirin in Reducing Events in the Elderly trial (ASPREE) put previous findings into question. The trial enrolled 19,114 patients, 70 years of age or older (median age, 74 years), without cardiovascular disease, dementia, or physical disability. Patients were randomized to receive 100 mg of enteric-coated aspirin daily or placebo. The primary endpoint was the onset of cardiovascular events. A significantly greater number of deaths was observed in the aspirin arm, driven by a 31% increase in cancer-related deaths. Cancer-related mortality occurred in 3.1% of aspirin users compared to 2.3% of aspirin nonusers (HR, 1.31; 95% CI, 1.10–1.56), within a median follow-up of 4.7 years. The higher cancer-related mortality in the aspirin group was not confined to a specific tumor location [13]. However, aspirin use was not strongly associated with higher cancer incidence in the ASPREE trial (981 cancers in the aspirin group compared to 952 cancers in the placebo) and results should be interpreted with caution. The effect of aspirin on cancer-related mortality observed in the ASPREE trial might be due to a decreased survival among participants with undiagnosed cancers at time of enrollment rather than a true effect of aspirin on cancer incidence and cancer-related mortality. In addition, the mean age of participants was approximately 60 years in the meta-analyses of RCTs comparing daily aspirin versus no aspirin use in the primary prevention of CVD while the ASPREE trial enrolled patients 70 years of age or older. In the most recent meta-analysis including results from the ASPREE trial, there was no significant difference in cancer-related mortality between aspirin users and aspirin non-users (HR, 1.03; 95% CI, 0.96–1.11). Results remained similar even after exclusion of the ASPREE trial (HR, 0.98; 95% CI, 0.91–1.06) [61].

Together, these data suggest that aspirin has no effect on overall cancer incidence and related mortality, except in CRC.

### 2.3. Benefit of Aspirin as Adjuvant Treatment in Patients with Cancer

An association between aspirin use in cancer patients and metastatic spread was first suggested by a long-term follow-up study of five large RCTS examining daily aspirin in the prevention of CVD (n = 17,285 participants; mean in-trial follow-up, 6.5 years). An incident in-trial solid cancer of known metastasis status occurred in 775 participants, and metastases were less frequent in the aspirin group compared to the control group (182/409 versus 211/366; OR, 0.59; 95% CI, 0.44–0.78; *p* = 0.0003), especially for CRC (OR, 0.36, 95% CI, 0.18–0.74; *p* = 0.005) [65]. Similarly, a recent systematic review and meta-analysis of all studies reporting aspirin use in cancer patients found that aspirin was associated with a significant 25% reduction in CRC-related mortality (HR, 0.75; 95% CI, 0.68–0.83), in a significant 20% reduction in breast cancer-related mortality (HR, 0.80; 95% CI, 0.66, 0.97) and in a 15% reduction in prostate cancer-related mortality (HR, 0.86; 95% CI 0.78–0.95). Pooled analysis of all studies reporting on metastatic spread suggested that aspirin reduced metastatic spread (five studies, RR, 0.77; 95% CI, 0.65, 0.92). However, these results should be interpreted with caution given the high heterogeneity between studies [66].

Direct evidence on the potential benefit of aspirin given as adjuvant treatment in cancer patients is limited to three small randomized-controlled trials, which were negative. A small trial examining patients with Duke’s B2 and CRC randomized 66 patients to receive 1200 mg aspirin per day or placebo as adjuvant treatment. No difference in progression-free survival or overall survival was found between the groups (HR for overall survival, 0.65; 95% CI, 0.02–18.06, *p* = 0.90) [67]. Lebeau et al. randomized 303 small cell lung cancer patients to receive 1 g aspirin per day for 18 months or no aspirin, in addition to chemotherapy. Once again, no difference in overall survival was observed between the two study arms (HR, 1.01; 95% CI, 0.81–1.27) [68]. Finally, Creagan et al. randomized 176 patients with advanced renal cell cancer to receive interferon-α, with or without 2400 mg aspirin per day, and found no evidence of a survival benefit in the aspirin group (HR, 0.91; 95% CI, 0.63–1.3) [69].

The potential therapeutic effects of aspirin when used as an adjuvant treatment in patients with cancer are being explored in several ongoing clinical trials (detailed in Table 3) including the Aspirin for Breast Cancer (ABC) trial (NCT02927249) [70], the Aspirin for Dukes C and high-risk B COLorecTal cancer (ASCOLT) trial (NCT00565708) [71], and the ADD-ASPIRIN trial (NCT02804815) [72].

### 2.4. Potential Mechanisms of Action by Which Aspirin Exerts Its Anticancer Effects 

The anticancer effect of aspirin is hypothesized to be mediated by several inter-related mechanisms [2,3,5,6,7].

A compelling body of evidence supports the role of platelets in cancer progression: platelets have been demonstrated to promote tumor growth, to induce tumor angiogenesis, to activate tumor invasion by inducing epithelial-mesenchymal transition via TGFβ/SMAD signaling, and to support metastasis by facilitating tumor cell arrest and attachment to the endothelium, extravasation and seeding [5]. By irreversibly inhibiting platelet functions, aspirin might interrupt this platelet-cancer crosstalk [5].

In addition, aspirin also decreases PGE2 synthesis through inhibition of COX2 in tumor cells, resulting in inhibition of Wnt signaling to beta catenin, a pathway involved in cellular proliferation, growth and survival [2,3,5].

COX-independent anticancer effects of aspirin have also been suggested, including modifications of NF-κB and RUNX1, induction of cancer cell apoptosis, reversal of tumor suppressor genes hypermethylation, downregulation of mutation-inducing DNA damage and acetylation of intracellular RNA [5].

## 3. The Chemoprotective Effect of Other Antiplatelet Agents

Other antiplatelet agents have been investigated in several animal models [4,5,74,75]. They are also expected to confer a clinical benefit in cancer prevention. However, evidence for their chemoprotective effects, alone or in combination with low-dose aspirin, is limited and inconclusive.

### 3.1. The Chemoprotective Effect of Other Antiplatelet Agents on the Risk of Incident Cancer

#### 3.1.1. P2Y12 Antagonists

##### Evidence from Epidemiological Studies

Epidemiological studies that have investigated the potential benefit of P2Y12 ADP receptor antagonists on cancer incidence are scarce. Soriano et al. first reported a significant 22% to 34% reduced risk of CRC in clopidogrel users in three nested-control studies based on data from a large validated UK primary care database [76]. This risk reduction was equivalent in magnitude to the one observed with low-dose aspirin, with consistent results across the three nested-control studies and across patient subgroups [76].

Using data from a large population-based cohort study (*n* = 183,912), Leader et al. compared the benefit of aspirin given as monotherapy to those of dual antiplatelet therapy (DAPT) with aspirin and clopidogrel [77]. Patients with a diagnosis of cancer within the first year were excluded from the analysis. Compared to subjects receiving no antiplatelet therapy, a 46% risk reduction in incident cancer was observed in aspirin users (adjusted HR, 0.54; 95% CI, 0.52–0.56; *p* < 0.001), and a 54% risk reduction was observed in DAPT users (adjusted HR, 0.46; 95% CI, 0.44–0.49; *p* < 0.001). Compared to aspirin given in monotherapy, DAPT resulted in an additional 8% risk reduction. This reduction was observed in patients with solid cancer (adjusted HR, 0.85; 95% CI, 0.79–0.92) and CRC (adjusted HR, 0.77; 95% CI, 0.64–0.92), but not in patients with hematological malignancies (adjusted HR, 0.93; 95% CI, 0.78–1.1) [77].

Recently, a nested case-control study of the Spanish population-based Base de datos para la Investigación Farmacoepidemiológica en Atención Primaria (BIFAP) study compared the chemoprotective effect of clopidogrel and low-dose aspirin on the risk of CRC [78]. The study included 15,491 incident cases of CRC and 60,000 controls matched by sex, age, and year of indexing. Clopidogrel (75 mg per day) used as monotherapy was significantly associated with a decreased risk of CRC overall (adjusted OR, 0.80; 95% CI, 0.69–0.93), similar in magnitude to the one observed with low-dose aspirin (adjusted OR, 0.83; 95% CI, 0.78–0.89). Risk reduction was more pronounced in subjects treated for 1 year (adjusted OR, 0.65; 95% CI, 0.55–0.78), and in those treated for 1 to 3 years (adjusted OR, 0.57; 95% CI, 0.45–0.73) [78]. A matched comparison of aspirin or clopidogrel given in monotherapy versus DAPT found that DAPT was not associated with any additional benefit. Beyond 1 year of continuous and current antiplatelet use, clopidogrel monotherapy offered the greatest risk reduction (37% versus 22% with aspirin monotherapy and versus 22% with DAPT) [78].

Finally, a retrospective registry study assessed the risk of de novo cancer diagnosis in acute coronary syndrome (ACS) patients discharged from a tertiary hospital with DAPT with either aspirin and clopidogrel, or aspirin and prasugrel, or aspirin and ticagrelor [79]. Among 4229 consecutive ACS patients, 311 were diagnosed with de novo cancer during a median follow-up of 46.2-months. The cumulative incidence of cancer was 2.2 per 100 patients per year for clopidogrel users, compared to 1.6 per 100 patients per year for prasugrel users, and 0.3 per 100 patients per year for ticagrelor users. In multivariate and propensity score analyses, ticagrelor use was associated with a lower risk of de novo cancer diagnosis compared to clopidogrel or prasugrel use (adjusted HR 0.22; 95% CI 0.05–0.90; *p* = 0.036), regardless of DAPT duration [79].

##### Evidence from Secondary Analyses of Randomized Controlled Trials of Dual Antiplatelet Therapy in the Secondary Prevention of Acute Coronary Syndrome

Secondary analyses of landmark RCTs of DAPT have generated controversies regarding the safety of long-term use of DAPT and the subsequent risk of cancer [80,81]. While early landmark RCTs assessing the efficacy and safety of the second generation thienopyridine clopidogrel given in monotherapy [82] or in DAPT [83,84,85] did not report any associated cancer risk, results of the Trial to Assess Improvement in Therapeutic Outcomes by Optimizing Platelet Inhibition with Prasugrel–Thrombolysis in Myocardial Infarction 38 (TRITON–TIMI 38) trial later suggested that DAPT with prasugrel, a third generation thienopyridine, may increase the risk of cancer. [86] Indeed, in TRITON-TIMI 38, adverse events related to cancer occurred more frequently in the prasugrel arm compared to the clopidogrel arm, with a difference emerging after four months of treatment, prompting additional post-hoc data collection. The post-hoc analysis of the trial indicated a trend for an increased risk of new cancer diagnosis in the prasugrel arm (1.6% versus 1.2% in the clopidogrel arm; RR, 1.29; 95% CI, 0.96–1.72) [87]. Moreover, in the DAPT trial [88], which assessed the impact of an extended duration of DAPT in patients with ACS undergoing percutaneous coronary intervention, a significant excess of cancer-related deaths was observed in patients receiving 30 months of DAPT, compared to those receiving 12 months of DAPT followed by aspirin plus placebo (31 versus 14, *p* = 0.02) [88], suggesting that DAPT should be limited to 1–2 years to avoid the potential risk of new cancers. However, this result was further attributed to an imbalance between groups at enrollment that might have affected the results (8 patients with cancer at enrollment in the thienopyridine group versus 1 patient in the placebo group) [81].

A pre-specified secondary analysis of the double-blind, randomized Targeted Platelet Inhibition to Clarify the Optimal Strategy to Medically Manage Acute Coronary Syndromes (TRILOGY ACS) trial [89] did not confirm the alarming finding from TRITON-TIMI 38. The study randomized 9326 ACS patients to receive DAPT with aspirin plus clopidogrel or aspirin plus prasugrel [90]. A new cancer occurred in 170 patients (1.8%) during the post-randomization follow-up period. In patients without a previous history of cancer or a previous curative treatment for cancer (*n* = 9105), the incidence of new cancer cases was similar in the prasugrel and in the clopidogrel groups (1.8 versus 1.7%; HR, 1.04; 95% CI 0.77–1.42; *p* = 0.79). The rate of bleeding was significantly higher in patients diagnosed with a new cancer compared to those without new cancer (53.5% versus 22.7%; *p* < 0.001).

Two meta-analyses found no association between thienopyridines and the risk of cancer or cancer-related mortality. A first systematic review and meta-analysis of 6 RCTs and 3 retrospective cohort studies evaluating the association between thienopyridine use and cancer found no association between thienopyridine use and the risk of new cancer (2 studies; OR, 0.92; 95% CI, 0.52–1.64) or cancer-related mortality (3 studies; OR, 1.12; 95% CI, 0.80–1.56) [91]. The results were consistent across sub-analyses according to cancer location. Compared with clopidogrel, prasugrel did not increase the risk of cancer onset (2 studies; OR, 1.10; 95% CI, 0.89–1.37) [91]. A second meta-analysis of 6 RCTs compared aspirin in combination with prolonged, short-duration, or no treatment with clopidogrel. The study found no association between clopidogrel use and the risk of cancer [92]. A new cancer occurred in 2.97% of clopidogrel users versus 2.96% of clopidogrel non-users (*p* > 0.99). No difference was observed between prolonged and short-duration treatment with clopidogrel [92].

#### 3.1.2. Thrombin Receptor Antagonists

The PAR-1 antagonist Vorapaxar was studied in two large Phase III randomized, double-blind, placebo-controlled clinical trials: the Thrombin Receptor Antagonist for Clinical Event Reduction in Acute Coronary Syndrome (TRACER) [93] and the Trial to Assess the Effects of Vorapaxar in Preventing Heart Attack and Stroke in Patients With Atherosclerosis-Thrombolysis In Myocardial Infarction (TRA2P) [94]. In TRACER [93], which compared vorapaxar to placebo, in combination with DAPT with aspirin and a P2Y12 inhibitor, in patients with ACS without ST-segment elevation, 27 deaths related to cancer occurred in the vorapaxar arm compared to 18 in the placebo arm (HR, 1.4; 95% CI, 1.1–1.8, *p* = 0.012). The time of first solid cancer development did not differ between the arms in TRA2P [80].

### 3.2. The Chemoprotective Effect of Other Antiplatelet Agents on the Risk of Cancer Progression

A single population-based cohort study assessed the benefit of clopidogrel on cancer recurrence. Hicks et al. examined the association between exposure to clopidogrel following cancer diagnosis and cancer-related mortality in a large prospective UK population-based cohort of newly diagnosed colorectal (*n* = 10,359), breast (*n* = 17,889) and prostate (*n* = 13,155) cancer patients [95]. After a 5-year average follow-up (range 0 to 13 years), the rate of cancer-related mortality did not differ between clopidogrel users and clopidogrel non-users, either for colorectal (adjusted HR, 0.96; 95% CI, 0.76–1.22) breast (adjusted HR, 1.24; 95% CI, 0.91–1.68) or prostate (adjusted HR, 1.03; 95% CI, 0.82–1.28) cancer patients, suggesting a neutral effect of clopidogrel on cancer progression.

## 4. Conclusions

Several organizations, such as the US Preventive Task Force guidelines, the US Multi-society Task Force on Colorectal Cancer and the National Institute for Health and Care Excellence, acknowledging the significant number of studies assessing the chemoprotective effects of aspirin and the resulting evidence, now recommend the routine use of aspirin for the primary prevention of CRC in some subgroups of patients for which the benefits are deemed to outweigh the risks. However, several questions still remain unanswered, such as the optimal aspirin dose and duration of treatment.

In contrast, the chemoprotective effects of aspirin against other cancers remains a controversial issue and prospective studies are warranted to yield more definitive conclusions. Similarly, the chemoprotective effects of other antiplatelets drugs, given alone or in combination with low-dose aspirin, still remain unclear and need to be further documented in both experimental and clinical studies.

Ongoing placebo-controlled trials of antiplatelets drugs in patients with precancerous lesions or cancer will provide new insights on their efficacy and safety in this setting.

## Figures and Tables

**Table 1:** Placebo-controlled trials of aspirin for the prevention of cardiovascular disease (CVD) ± cancer reporting colorectal cancer (CRC) incidence and CRC-related mortality.

Trial (Reference)	Study Population	Number of Subjects Randomly Assigned	Treatment Groups	Median Treatment Duration (Years)	Primary End Point	RR (95% CI)	
						CRC Incidence	CRC-Related Mortality
PHS [19]	Male physicians (age 40–84 years) without history of MI, stroke, cancer, liver or renal disease, gout, peptic ulcer or contraindications to aspirin	22,071	Placebo versus 325 mg aspirin alternate day	5.0	CVD and cancer	1.03 (0.83–1.28)	Not reported
WHS [21]	Women (age ≥ 45 years) without history of cancer (except non-melanoma skin cancer), CVD or other major chronic illness	39,876	Placebo versus 100 mg aspirin alternate day	10.1	CVD and cancer	0.80 (0.67–0.97)	0.80 (0.67–0.97)
BDA [22]	Male physicians without peptic ulcer, stroke or definite MI	5139	Placebo versus 500 mg aspirin per day	6.0	CVD	0.70 (0.51–0.97)	0.73 (0.49–1.10)
UK-TIA Aspirin Trial [23]	Patients with prior TIA or stroke	2449	Placebo versus 300 or 1200 mg aspirin per day	4.4	CVD	0.75 (0.56–0.97), [25] pooled with TPT and SALT	0.61 (0.43–0.87), [25] pooled with TPT and SALT
TPT [41]	High risk for IHD	5085	Placebo versus 75 mg aspirin per day (alone or with warfarin)	6.9	CVD	0.75 (0.56–0.97), [25] pooled with UK-TIA and SALT	0.61 (0.43–0.87), [25] pooled with UK-TIA and SALT
SALT [42]	Prior TIA or stroke or retinal occlusion	1363	Placebo versus 75 mg aspirin per day	2.7	CVD	0.75 (0.56–0.97), [25] pooled with TPT and UK-TIA	0.61 (0.43–0.87), [25] pooled with TPT and UK-TIA

BDA, British Doctors Aspirin Trial; CI, confidence interval; CRC, colorectal cancer; CVD, cardiovascular disease; IHD, Ischemic heart disease; PHS, Physicians’ Health Study; RR, relative risk; SALT, Swedish Aspirin Low Dose Trial; TIA, Transient Ischaemic Attack; TPT, Thrombosis Prevention Trial; UK-TIA, UK-Transient Ischaemic Attack; WHS, Women’s Health Study.

**Table 2:** Placebo-controlled trials of aspirin for the prevention of recurrent adenoma and colorectal cancer (CRC).

Trial (Reference)	Study Population	Number of Subjects Randomly Assigned	Treatment Groups	Median Follow-Up Duration (Years)	Primary End Point	RR (95% CI)	
						Any Adenoma	Advanced ADENOMA
AFPPS [33]	Recent history of resected sporadic colorectal adenoma	1121	Placebo versus 81 mg aspirin per day versus 325 mg aspirin per day, with or without folic acid	3.0	Recurrent adenoma	0.88 (0.77–1.02)	0.74 (0.52–1.06)
CALGB [34]	Previous resection of Dukes’ stage A or B1 CRC or B2 CRC and 5-year disease-free survival	635	Placebo versus 325 mg aspirin per day	1.1	Adenoma	0.61 (0.44–0.86)	0.77 (0.29–2.05)
APACC [36]	Recent history of sporadic colorectal adenomas	272	Placebo versus 160 mg aspirin per day versus 300 mg aspirin per day	4.0	Recurrent adenoma	0.95 (0.75–1.21)	0.91 (0.51–1.60)
ukCAP [35]	Recent history of resected sporadic colorectal adenomas	939	Placebo versus 300 mg aspirin per day	3.0	Recurrent adenoma	0.79 (0.63–0.99)	0.63 (0.43–0.91)
J-CAPP [38]	Recent history of resected colorectal adenomas and CRCs	311	Placebo versus 100 mg aspirin per day with or without folate supplement	2.0	Recurrent adenoma and CRC	0.60 (0.36–0.98)	
Chemoprevention of Colorectal Adenomas [39]	Recent history of resected sporadic colorectal adenomas	1107	Placebo versus 75 mg aspirin per day with 1,25-dihydroxycholecalciferol + calcium	3.0	Recurrent adenoma	0.95 (0.61–1.48)	RR not reported
seAFOod [40]	Recent history of resected sporadic colorectal adenomas	709	Placebo versus 2 g EPA-free fatty acid (FFA) per day or 300 mg aspirin per day or both treatments in combination	1.0	Recurrent adenoma	0.99 (0.87 to 1.12) risk difference = –0.6% (95% CI, –8.5 to 7.2) *p* = 0.88	RR not reported risk difference = −0.3% (95% CI, −4.1 to 3.5)

AFPPS, Aspirin/Folate Polyp Prevention Study; APACC, Association pour la Prévention par l’Aspirine du Cancer Colorectal; CALGB, Colorectal Adenoma prevention study originated in the cooperative trials group cancer and Leukaemia Group B; CI, confidence interval; CRC, colorectal cancer; J-CAPP, Japan Colorectal Aspirin Polyps Prevention; RR, relative risk; seafood, Systematic Evaluation of Aspirin and Fish Oil; ukCAP, United Kingdom Colorectal Adenoma Prevention.

**Table 3:** Ongoing placebo-controlled trials of aspirin in patients with precancerous lesion or with cancer.

Trial (Reference)	Study Population	Estimated Enrollement	Treatment Groups	Treatment Duration (Years)	Primary End Point	End Date
ABC trial [70] (NCT02927249)	Early stage node positive HER2 negative breast cancer patients	2936	Placebo versus Aspirin 300 mg per day	5.0	5-years invasive disease-free survival	2021
ASCOLT [71] (NCT00565708)	Dukes C or high-risk Dukes B colorectal cancer who have completed surgery and standard adjuvant chemotherapy	1587	Placebo versus Aspirin 200 mg per day	5.0	5-years disease free survival	2025
ASPIRED [73] (NCT02394769)	previously diagnosed with colorectal adenoma	180	Placebo versus Aspirin 81 or 325 mg per day	12 weeks	Molecular biomarkers including urinary prostaglandin metabolites (PGE-M; primary endpoint), and specific biomarkers of colorectal carcinogenesis	2029
ADD-ASPIRIN [72](NCT02804815)	Breast Cancer Prostate Cancer Colorectal Cancer Gastro-oesophageal Cancer	11,000	Placebo versus Aspirin 100 or 300 mg per day	5.0	5- and 10-years overall survival 6-years invasive disease-free survival 6-years disease-free survival 5-years biochemical recurrence-free survival (bRFS)	2026

ABC, Aspirin for breast cancer; ASCOLT, Aspirin for Dukes C and high-risk B COLorecTal cancer; ASPIRED (ASPirin Intervention for the REDuction of Colorectal Cancer Risk); ADD-ASPIRIN, A Trial Assessing the Effects of Aspirin on Disease Recurrence and Survival After Primary Therapy in Common Non-metastatic Solid Tumours.
